# Transcriptome Sequencing and Chemical Analysis Reveal the Formation Mechanism of White Florets in *Carthamus tinctorius* L.

**DOI:** 10.3390/plants9070847

**Published:** 2020-07-04

**Authors:** Tingyan Qiang, Jiushi Liu, Yuqing Dong, Yinbo Ma, Bengang Zhang, Xueping Wei, Haitao Liu, Peigen Xiao

**Affiliations:** 1 Institute of Medicinal Plant Development, Chinese Academy of Medical Sciences & Peking Union Medical College, Beijing 100193, China; qiangtingyan@163.com (T.Q.); jsliu@implad.ac.cn (J.L.); yuqingdong97@163.com (Y.D.); bgzhang@implad.ac.cn (B.Z.); xpwei@implad.ac.cn (X.W.); pgxiao@implad.ac.cn (P.X.); 2 Molecular Genetics and Genomics Laboratory, Department of Horticulture, Chungnam National University, Daejeon 34134, Korea; mayinbo@126.com

**Keywords:** *Carthamus tinctorius* L., white color, safflower pigments, transcriptome sequencing, chemical analysis

## Abstract

*Carthamus tinctorius* L. (safflower), an economic crop and herb, has been extensively studied for its diverse chemical constituents and pharmacological effects, but the mechanism of safflower pigments (SP) leading to different colors of florets has not been clarified. In the present study, we compared the contents of SP in two varieties of safflower with white and red florets, named Xinhonghua No. 7 (WXHH) and Yunhong No. 2 (RYH). The results showed the contents of SP in RYH were higher than WXHH. To investigate genes related to SP, we obtained six cDNA libraries of florets from the two varieties by transcriptome sequencing. A total of 225,008 unigenes were assembled and 40 unigenes related to safflower pigment biosynthesis were annotated, including 7 unigenes of phenylalanine ammonia-lyase (PAL), 20 unigenes of 4-coumarate-CoA ligase (4CL), 1 unigene of trans-cinnamate 4-monooxygenase (C4H), 7 unigenes of chalcone synthase (CHS), 4 unigenes of chalcone isomerase (CHI), and 1 unigene of flavanone 3-hydroxylase (F3H). Based on expression levels we selected 16 differentially expressed unigenes (DEGs) and tested them using reverse transcription-quantitative real-time polymerase chain reaction (RT-qPCR), which was consistent with the sequencing results. Consequently, we speculated that in WXHH, 3 *PALs*, 3 *4CLs*, 1 *C4H*, 1 *CHS*, and 1 *CHI*, which were down-regulated, and 1 *F3H*, which was up-regulated, may play a key role in the formation of white florets.

## 1. Introduction

*Carthamus tinctorius* L., known as safflower, is the only species of *Carthamus* (Asteraceae) distributed in China [1], and it is native to Northern India and the Asiatic regions of the Middle East [2]. *Carthami flos* recorded in Chinese Pharmacopoeia (2015 edition) is the dried flowers of safflower, which are collected in summer when their colors change from yellow into red [3]. Phytochemisty research demonstrates that there are more than 200 compounds detected and isolated from *Carthami flos*, such as quinochalcones, flavonoids, lignans, alkaloids, fatty acids, sesquiterpenes, polyacetylenes, sterols, and polysaccharides [4,5,6]. Quinochalcone C-glycosides (QCGs) are the characteristic and chief active compounds [7]. Modern pharmacological studies indicate *Carthami flos* possesses a wide range of biological activities, such as anti-inflammatory and analgesic, antithrombotic, antioxidant, hepatoprotective, antidiabetic, etc. [8,9,10]. Meanwhile, safflower is one of the most ancient crops and was first used commercially in China more than 2200 years ago [11]. In addition to the ornamental value [12], the flowers of safflower can be used to extract pigments which can be used in food, textile, and cosmetic [13] and the seeds can yield edible oil (about 35–45% of the seed oil) [14,15,16]. Therefore, safflower has potential to be developed for its flowers and seeds.

**Figure 1:**
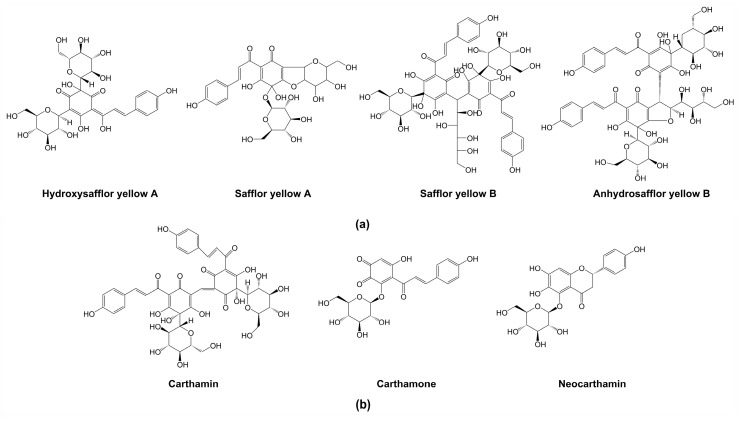
(**a**) Chemical structures of four respective compounds of safflower yellow pigments (SYP); (**b**) chemical structures of three compounds of safflower red pigments (SRP).

Since safflower was introduced by Zhang Qian in the historic “Silk Road” [9], the regional environment differences had made the safflower variety rich and diverse [17], such as varieties with different colors of florets. It is reported that the attractive natural display of flower colors is attributable to anthocyanins, betalains, and carotenoids [18,19,20]. However, safflower pigments (SP) are unique in safflower flowers, which include water-soluble yellow pigments and water-insoluble red pigments [21,22]. The content of safflower yellow pigments (SYP) in safflower is about 20–30%, while the content of safflower red pigments (SRP) is extremely low, only accounting for about 0.3–0.6% [23]. SYP consists of many compounds, in which 80–90% is hydroxysafflor yellow A (HSYA) [24]. However, at present only three SRP compounds, carthamin, neocarthamin, and carthamone have been found, of which carthamin is the main component [25,26,27]. All compounds of SYP, carthamin, and carthamone are classified into the quinochalcone family of flavonoids, which have a unique structure with a C-glycosylated cyclohexanonedienol moiety that occurs only in safflower (Figure 1) [28,29,30]. However, neocarthamin belongs to flavanone (Figure 1) [31].

With the rapid development of RNA-Seq, several studies on the molecular mechanism of pigments have been conducted, such as grape hyacinth, tropical waterlily, potato, etc. [32,33,34]. As for safflower, there have been several investigations on the transcriptome, especially the biosynthesis of flavonoids. For example, the genes encoding UDP-glycosyl-transferase [35], flavanone 3-hydroxylase [36], chalcone synthase [37] and chalcone isomerase [38] in safflower have been cloned and verified. Furthermore, the putative genes associated with safflower yellow biosynthesis in safflower were investigated [39]. However, the biosynthesis pathway and candidate genes related to the formation of both SYP and SRP have not been studied comprehensively due to their special structures and properties.

Flavonoids are widely distributed secondary metabolites with different metabolic functions in plants and have attracted widespread attention in recent years [40]. Flavonoids include six major subgroups found in most higher plants: chalcones, flavones, flavonols, flavandiols, anthocyanins, and condensed tannins (or proanthocyanidins); a seventh group, the aurones [41]. Over the years, the role of flavonoids as pigments conferring colors to plants has attracted widespread attention to their structures, properties, biosynthesis, and biological functions [42,43]. It is generally known that flavonoids are synthesized through the phenylpropanoid pathway, transforming phenylalanine into *p*-coumaroyl-CoA, which finally enters the flavonoid biosynthesis pathway [44]. Concretely, phenylalanine ammonia-lyase (PAL) catalyzes phenylalanine and tyrosine to generate cinnamic acid and *p*-cinnamic acid, respectively. Cinnamic acid can be catalyzed by trans-cinnamate 4-monooxygenase (C4H) to generate *p*-cinnamic acid. Then both cinnamic acid and *p*-cinnamic acid can be catalyzed by 4-coumarate-CoA ligase (4CL) to generate cinnamoyl-CoA and *p*-cinnamoyl-CoA, and the former can also be catalyzed by C4H to generate the latter [45]. Then, the first enzyme chalcone synthase (CHS) in flavonoid biosynthesis [40], a key and rate-limiting enzyme [46], can catalyze 1 molecule *p*-cinnamoyl-CoA and 3 molecules malonyl-CoA to produce naringenin chalcone. Chalcone isomerase (CHI) can convert naringenin chalcone into naringenin, which can be converted by flavanone 3-hydroxylase (F3H) into dihydrokaempferol.

In the present study, the first RNA-Seq project for the white florets of Xinhonghua No. 7 (WXHH) and the red florets of Yunhong No. 2 (RYH) was conducted using the Illumina Hiseq Sequencing platform and was analyzed. In addition, we compared the contents of SYP and SRP in the two varieties of safflower. By combining transcriptome sequencing with chemical analysis, the contents of SYP and SRP, which lead to the phenotypes of red and white florets directly, were compared and interpreted. In addition, the major metabolic pathway related to safflower pigment biosynthesis was deduced and the candidate genes leading to the formation of SP were predicted and validated by reverse transcription-quantitative real-time polymerase chain reaction (RT-qPCR). This study provided a foundation for further studies on SP to find important genes which can help in producing a high yield of SYP and SRP whether through plant breeding or metabolic engineering.

## 2. Results

### 2.1. Contents of Safflower Yellow Pigments (SYP) and Safflower Red Pigments (SRP)

Quantitative analysis of SYP was conducted by external standard method, took the absorbance value ‘A’ of each HSYA reference solutions at 403 nm as the abscissa, and the concentration ‘C’ (Unit: mg/mL) of each solution as the ordinate, the regression equation of the standard curve was obtained as followed: C = 0.0288A − 0.0006, R^2^ = 0.9995. The content of SYP in each sample was obtained by substituting its absorbance value at 403 nm into the above regression equation. Due to the lack of reference materials of SRP, the contents of SRP in the six samples were compared by using the absorbance values at 520 nm. As shown in Table 1, both the contents of SYP and SRP were higher in RYH than in WXHH. The average content of SYP in RYH was more than 7 times as much as that in WXHH. Furthermore, the average ‘content’ of SRP in RYH was more than 30 times as much as that in WXHH.

### 2.2. De Novo Transcriptome Assembly and RNA-Seq Analyses

In order to interpret the mechanism of flower color variation in safflower, six cDNA libraries (W1, W2, W3, R1, R2, R3) were constructed. The six libraries were sequenced by using an Illumina Hiseq Sequencing platform with 2 × 150 bp paired-end reads. A total of 19.1 GB raw data were obtained (NCBI accession: PRJNA590138). After removing the adaptor sequence, low-quality reads, short reads, and sequence with uncertain base information, a total of 277,503,770 clean reads were obtained (141,468,302 and 136,035,468 clean reads of RYH and WXHH samples, respectively). The average Q20 percentage (percentage of sequences with sequencing error rates < 1%) of the two groups were 98.95% and 98.97%, respectively. These data showed that the RNA-Seq quality was high enough for further analysis (Table 2). Subsequently, de novo assembly generated a total of 225,008 unigenes with an average length of 697.36 bp, with the N50 (covering 50% of all the nucleotide sequences of the largest unigene length) of 1080 bp. In total, 159,524 unigenes (71%) were between 0 and 500 bp; 38,644 unigenes (17%) were between 501 and 1000 bp. The results of aligning clean reads of each sample to unigenes are given in Table S1.

### 2.3. Functional Annotation and Classification

**Figure 2:**
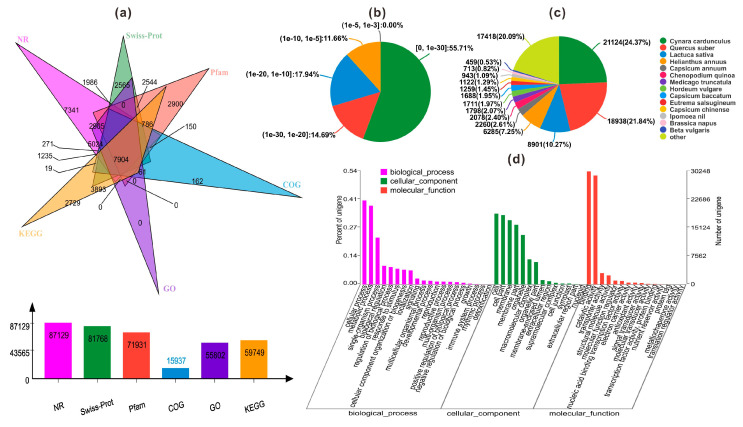
(**a**) Summary of the annotations for the assembled unigenes in six public databases; Characteristics of homology search of two varieties of safflower unigenes; (**b**) *E*-value distribution of the top BLASTx hits against the NR database for each unigene; (**c**) Number and percentage of unigenes matching the top 15 species using BLASTx in the NR database; (**d**) Gene Ontology (GO) categories assigned to the two varieties of safflower.

Based on the de novo assembly of the transcriptome database, only 109,360 unigenes accounting for 48.6% of total unigenes were annotated to six databases, including NR (NCBI non-redundant protein sequences), Swiss-Prot, Pfam, COG (Clusters of Orthologous Groups of proteins), GO (Gene Ontology), and KEGG (Kyoto Encyclopedia of Genes and Genomes) as shown in Figure 2a. Among them, 87,129 unigenes (38.72% of all annotated unigenes) could be annotated to the NR database, while 81,768 (36.34%), 71,931 (31.97%) and 15,937 (7.08%) were annotated to Swiss-Prot, Pfam, and COG. And we annotated 55,802 (24.8%) and 59,749 (26.55%) unigenes to GO and KEGG databases.

According to NR annotation and *E*-value distribution, 70.4% of the annotated unigenes had strong homology (*E*-value < 10^−20^) and 55.71% of the annotated unigenes had very strong homology (*E*-value < 10^−30^) (Figure 2b). The 15 top-hit species based on NR annotation are shown in Figure 2c. Nearly 65% of unigenes were annotated with the sequences from the 4 top-hit species, *Cynara cardunculus*, *Quercus suber*, *Lactuca sativa* and *Helianthus annuus*. Among these, *Cynara cardunculus* had the highest homology with annotated unigenes accounting for 24.37%. In addition, the results of GO annotation was shown in Figure 2d, including the cellular component (CC), molecular function (MF), and biological process (BP).

### 2.4. Identification and Functional Enrichment Analysis of Differentially Expressed Genes (DEGs)

**Figure 3:**
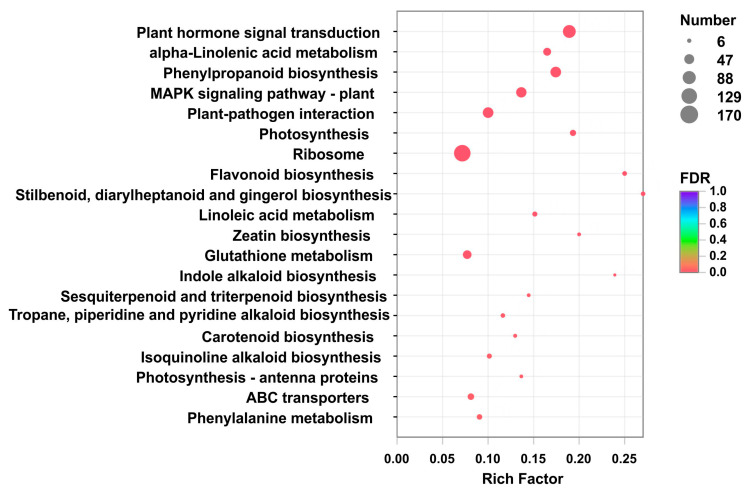
Pathway enrichment analysis by comparison between WXHH and RYH. The ratio between the number of differentially expressed genes (DEGs) mapped to a pathway and the total number of genes mapped to that pathway are indicated by enrichment factors. The vertical axis represents the pathway name, and the horizontal axis represents the rich factor. The larger the rich factor is, the greater the degree of enrichment will be. The size of the point represents the number of unigenes in this pathway, and the color of the point corresponds to different *p*-value range.

**Figure 4:**
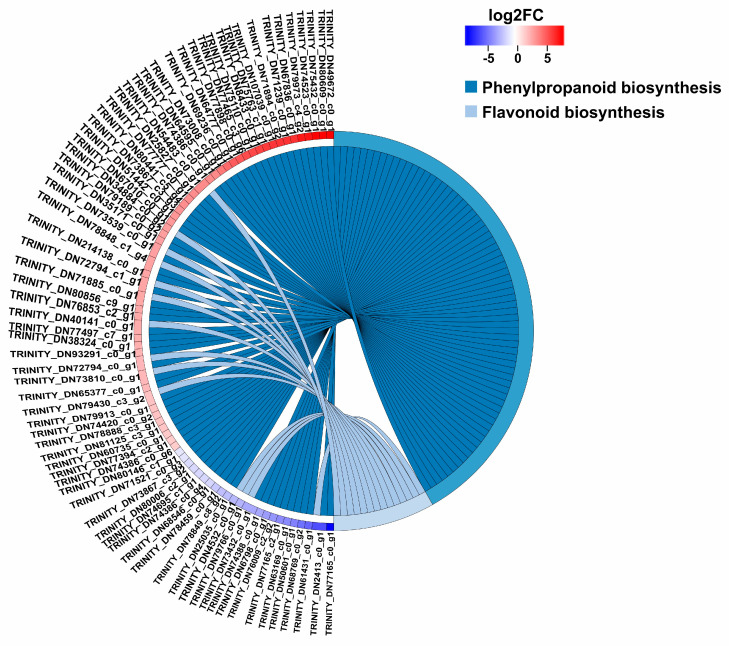
85 DEGs mapped in the two Kyoto Encyclopedia of Genes and Genomes (KEGG) pathways (map00940 and map00941) related to safflower pigment biosynthesis.

A total of 8725 differentially expressed genes (DEGs) (*p*-value < 0.05 & |log2(FC)| >= 1) were obtained by comparing W1, W2, W3 with R1, R2, R3, among which 4970 were down-regulated as shown in Figure S1. Based on the number of DEGs mapping to a pathway/total number of unigenes mapped to the pathway (enrichment factors), which could estimate the relative degree of enrichment in these pathways, the top 20 KEGG pathways significantly (*p*-value < 0.05) enriched were obtained as shown in Figure 3. The maximum enrichment factor (0.27) for pathways in the 8725 DEGs was stilbenoid, diarylheptanoid and gingerol biosynthesis (map00945), followed by flavonoid biosynthesis (0.25, map00941), indole alkaloid biosynthesis (0.24, map00901), zeatin biosynthesis (0.20, map00908), photosynthesis (0.19, map00195), plant hormone signal transduction (0.18, map04075), and phenylpropanoid biosynthesis (0.17, map00940). Because the biosynthesis of safflower pigments refers to both phenylpropanoid biosynthesis (map00940) and flavonoid biosynthesis (map00941), 85 DEGs involved in the two pathways were focused (Figure 4 and Figures S2 and S3).

### 2.5. Genes Involved in the Safflower Pigment Biosynthesis

**Figure 5:**
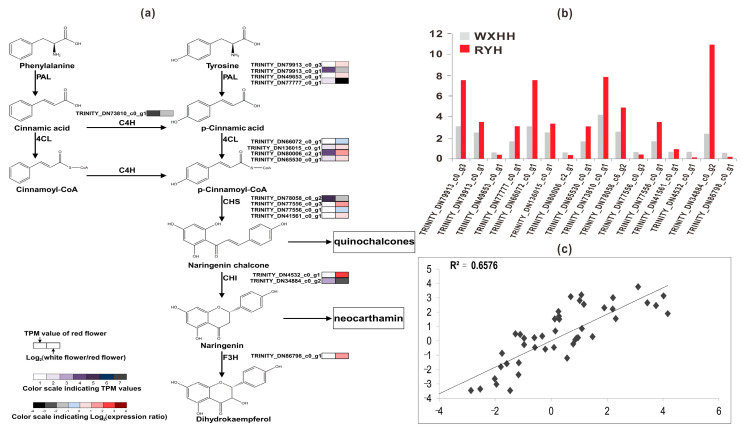
Schematic of physiological data related to flower color development of safflower. (**a**) A detailed part of safflower pigments (SP) metabolic subnetwork showing the subset of nodes or metabolites that constitute the process, (**b**) Expression level of colour-related unigenes measured by reverse transcription-quantitative real-time polymerase chain reaction (RT-qPCR), (**c**) the correlation analysis between RNA-seq and RT-qPCR.

Based on KEGG Orthology (KO) id and gene name or synonyms as shown in Table 3, we found out a total of 40 unigenes, including 7 *PAL*s, 20 *4CL*s, 1 *C4H*, 7 *CHS*s, 4 *CHI*s, and 1 *F3H*. Among them, based on expression level we selected 16 possible unigenes for verification via RT-qPCR (Table S2). The results showed that transcriptome sequencing data were credible because the correlation coefficient between the transcriptome sequence and RT-qPCR was high (R^2^ = 0.6576) (Figure 5c). In addition, the results of gene structure analysis of the 16 unigenes, including coding sequence (CDS) prediction, single nucleotide polymorphisms (SNP) analysis, and simple sequence repeats (SSR) analysis were shown in Table S3.

### 2.6. Characterization of 4 Chalcone Synthase (CHS) Unigenes

**Figure 6:**
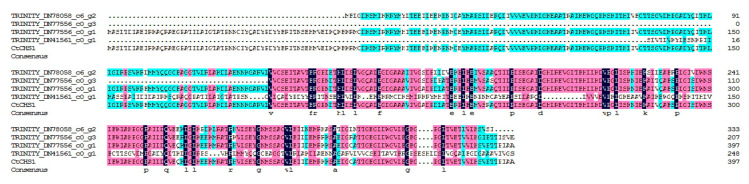
Alignment of deduced amino acid sequences of 4 chalcone synthase (CHS) unigenes and *CtCHS1*.

CHS is an enzyme which plays an important role in the synthesis of flavonoids. However, by now, the function of CHS is not yet clear in the biosynthesis of safflower flavonoids. BLASTX analysis of 4 CHS unigenes against the *CtCHS1* (KY471385) [37] showed that the deduced amino acid of TRINITY_DN77556_c0_g1 showed the highest identity to *CtCHS1* as shown in Figure 6. Phylogenetic analysis (Figure S4) showed that TRINITY_DN77556_c0_g1 has 100% similarity with *CtCHS1* from safflower, which meant the amino acid activity of it has certain similarity to *CtCHS1* which can increase the accumulation of quinochalcone. In addition, the other 3 CHS unigenes were less closely related to *CtCHS1*, which suggested that the 4 CHS proteins may possess different biological functions in safflower. To verify further the differential expression of TRINITY_DN77556_c0_g1, RT-qPCR was carried out and confirmed it has a higher transcript level at RYH than WXHH (Figure 5b).

### 2.7. Candidate Genes Responsible for the Decrease of Safflower Pigments (SP) in Xinhonghua No. 7 (WXHH)

As shown in Figure 5a,b, a total of 16 possible unigenes including 4 *PAL*s, 4 *4CL*s, 1 *C4H*, 4 *CHS*s, 2 *CHI*s, and 1 *F3H* were verified. Among them there were 3 *PAL*s, 3 *4CL*s, and 1 *C4H* down-regulated in WXHH, which were thought to be the flux-limiting unigenes leading to the decrease of SP in WXHH. Because of the characterization of 4 CHS unigenes, we concluded TRINITY_DN77556_c0_g1 may play a key role in quinochalcone biosynthesis rather than flavanone biosynthesis, and its low transcription level was the cause of the low content of SP in WXHH. In addition, CHI can convert naringenin chalcone into naringenin, which is the precursor of neocarthamin (SRP). There was 1 unigene of CHI-like sequence, TRINITY_DN34884_c0_g2, which had more than 7 times higher level of gene transcripts in RYH than in WXHH. Consequently, we deduced this unigene played a key role in neocarthamin biosynthesis rather than dihydroflavonol biosynthesis. Additionally, there was 1 unigene of F3H-like sequence, which had more than 3 times higher level of gene transcripts in WXHH than in RYH. It could be inferred that when *F3H* was up-regulated, the substrate naringenin used for neocarthamin synthesis was catalyzed to form dihydrokaempferol instead, which resulted in extremely low content of naringenin in WXHH.

## 3. Discussion

In recent years, with the increasing public awareness of the toxicity of synthetic dyes used as additives, the synthetic dye industry has declined and people are now focusing attention on natural pigments [47,48]. Furthermore, color, which is one of the sensory evaluation indexes for TCM (Traditional Chinese Medicine) and has been used for centuries, can reveal the internal quality of TCM and has been recognized as an important parameter by the Chinese Pharmacopeia [49]. And it’s recorded in ancient Chinese books of material medica that the red safflower is better in quality than other colors. Hence, the yield and color values of SP are important indicators for evaluating the quality of safflower germplasm. SP cannot only be used as color additives in food, textile, and cosmetics, but also have medicinal value on cardiac diseases, diabetes mellitus, hypertension, and so on [50]. In addition, compared with other colorants, SYP have many advantages, including being highly soluble in water, cheap, stable to light in aqueous solution, and they can be used at different temperatures and pH values [51]. SRP can be made into a dye with highly appreciated shades of pink, which are mainly used in lipstick, rouge, and other high-grade cosmetics. Consequently, in consideration of the medicinal and commercial importance of SP, the demand for safflower florets to extract SP is rising around the world. However, the current yield, mainly stemming from Asia and North America, is far from enough to meet the increasing demand. Therefore, it is necessary to increase the production of SP.

Safflower is an annual herbaceous plant adapting mainly to dry land and saline conditions. The growth and yield of safflower are affected by many factors such as genotype, environment, and agronomic measures [52]. The abundant genetic and phenotypic diversity of germplasm resources is the foundation to promote the development of crop improvement strategies [53]. Consequently, in the present study the first transcriptome sequencing for two varieties of safflower with red and white florets was conducted in order to explore the possible genes to determine the yield of SP directly. A total of 225,008 unigenes were obtained, which is a valuable resource for further studies on the secondary metabolite biosynthesis of safflower. Subsequently, through annotation, differential expression analysis, functional enrichment analysis, validation, and characterization analysis, several insights into the formation mechanism of white florets of safflower were obtained. A total of 16 candidate unigenes including 4 *PAL*s, 4 *4CL*s, 1 *C4H*, 4 *CHS*s, 2 *CHI*s, and 1 *F3H* were selected and verified via RT-qPCR. The results of RT-qPCR were in accordance with transcriptome sequencing data (R^2^ = 0.6576). Based on the results of RT-qPCR, we inferred that 3 *PAL*s, 3 *4CL*s, 1 *C4H*, 1 *CHS*, and 1 *CHI*, which were down-regulated in samples of WXHH compared with RYH, may be the flux-limiting unigenes leading to the decrease of SP. And 1 up-regulated *F3H* in WXHH led to the extremely low content of neocarthamin in WXHH. However, there were a few unexpected unigenes, including 1 *PAL* (TRINITY_DN49653_c0_g1), 1 *4CL* (TRINITY_DN80006_c2_g1), 1 *CHS* (TRINITY_DN77556_c0_g3), and 1 *CHI* (TRINITY_DN4532_c0_g1), which were up-regulated in WXHH, we considered that if these reactions were all extremely suppressed in WXHH, not only SP but also other classes of flavonoids would decrease.

At present some major agronomic traits of safflower have been taken as main selection targets, but insufficient attention has been paid to the important characteristic of color value, which has resulted in uneven pigment contents in safflower varieties and varieties with high color value are lacking [23]. In the present study, we expounded on the possible molecular mechanisms that regulate the safflower pigment biosynthesis pathway. Furthermore, the transcriptomic data of WXHH and RYH generated in this study will be a significant resource for further studies on safflower, such as functional genomics, molecular biology, metabolic engineering, and plant breeding.

## 4. Materials and Methods

### 4.1. Plants Materials

**Figure 7:**
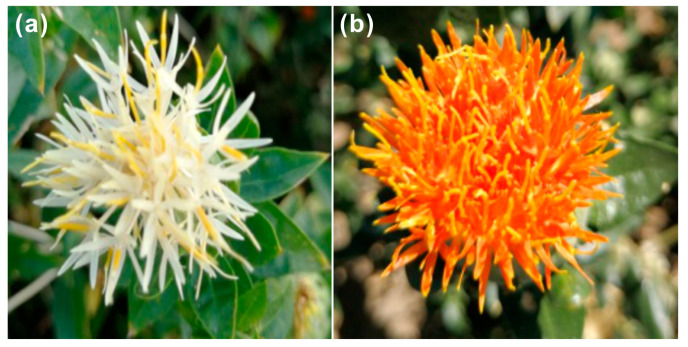
Safflower flower phenotypes of different varieties. (**a**) WXHH, (**b**) RYH.

The first flowers of safflower were collected at the same time from different plants grown in environmental conditions with permission in August 2018 from fields in Yumin County, Tacheng District, Xinjiang Uygur Autonomous Region, China (Figure 7a,b), and the seeds were obtained from Tacheng Agricultural Technology Extension Station. We took the capitula of each safflower flower, three capitula were used per replicate, with a total of three replicates collected from different plants (Table S4). The capitula were picked and immediately frozen in liquid nitrogen and stored at −80 ℃ for RNA extraction. In addition, the air-dried flowers from the same six batches of safflower were pulverized into powder, passed through a 40-mesh sieve and stored at room temperature for chemical analysis.

### 4.2. Preparation of Reference Solutions

Reference compound, hydroxysafflor yellow A (HSYA) was purchased from Phytomarker Ltd., Tianjin, China. The mother liquor of HSYA was prepared by weighing 2.7 mg of HSYA accurately and fixing the volume of water in a 10 mL volumetric flask to obtain the reference solution with a concentration of 0.27 mg/mL. Then accurately transfer 0.1, 0.2, 0.4, 0.6, and 1.0 mL mother liquor into 10 mL colorimetric tubes with stoppers separately, and fix the volume with water. However, at present there are few SRP products on the market due to low content in safflower flowers and the high price of *Carthami flos*.

### 4.3. Preparation of Samples and Content Analysis

According to the properties of SYP and SRP, the extraction procedure was carried out as described previously [54]. The optimal extraction process of SYP by warm immersion method was as follows: powdered samples (1.0000 g) were suspended in 14 mL water in a 50 mL centrifuge tube, with a first pre-soaking time of 70 min, and then extracted for two cycles (50 min each) in 60 ℃ water bath. Then centrifuged and separated the supernatant. The safflower residue after the extraction of SYP was used to extract SRP. The extraction method was optimized as followed: 15 mL 80% ethanol was added to the residue, then it was extracted twice in a 45 ℃ water bath for 10 min each time. Similarly, centrifuged and separated the supernatant.

Because both SYP and SRP are mixtures and there may be compounds that have not been separated and identified from safflower flowers, we chose ultraviolet–visible (UV-Vis) spectrometry for content analysis, which is a method recorded in Chinese Pharmacopoeia (2015 edition) to determine the absorbance of substances within the wavelength range of 190–800 nm. Quantitative analysis of SYP was conducted by the external standard method based on the standard curve of HSYA with known concentrations. However, due to the lack of a reference compound, the contents of SRP extracted from six samples were compared based on the absorbance values of each sample at 520 nm.

### 4.4. RNA Extraction, Library Construction, and RNA Sequencing

Total RNA was extracted from pooled flower samples using a TRIzol® kit (Invitrogen, Carlsbard, CA, USA). RNA concentration and quality were evaluated using 2100 Bioanalyser (Agilent Technologies, Inc., Santa Clara, CA, USA) and ND-2000 (NanoDrop Thermo Scientific, Wilmington, DE, USA). High-quality RNA samples (OD260/280 = 1.8~2.2, OD260/230 ≥ 2.0, RIN ≥ 8.0, 28S:18S ≥ 1.0, > 2 μg) were used to construct the sequencing library. By adding fragmentation buffer, mRNA was broken into short fragments with a length of about 300 bp, which were taken as templates to synthesize cDNA. Then, PCR amplification was performed to enrich cDNA libraries. At last, the six libraries were sequenced in a single lane on an Illumina Hiseq sequencer (Illumina, San Diego, CA, USA) for 2 × 150 bp paired-end reads.

### 4.5. De Novo Transcriptome Assembly and Annotation

Clean reads were obtained from six cDNA libraries after removing the adapter sequences, low-quality sequences, unknown reads (N percentage > 10%) and ribosomes RNA by using SeqPrep [55] and Sickle [56] with default parameters. De novo transcriptome assemblies of the clean reads were performed separately using the assembler Trinity v2.8.5 [57]. Unigenes were generated by removing the redundant sequences and short transcripts. Then the unigenes were compared against the NR, Swiss-Prot, and COG databases to retrieve protein functional annotations using Diamond with a threshold E-value of 1 × 10^−5^. Metabolic pathway assignments to KEGG by using KO-Based Annotation System (KOBAS) v2.1.1 [58] was to understand the complex biological functions of the gene products. Blast2GO v2.5.0 (BioBam, Valencia, Spain) [59] and Hmmer v3.2.1 (Howard Hughes Medical Institute, Baltimore, MD, USA) [60] with default parameters was used to compare unigenes against the GO and Pfam databases.

### 4.6. Differential Expression and Functional Enrichment Analysis

Bowtie2 v2.3.5.1 (Johns Hopkins University, Baltimore, MD, USA) [61] was used to map reads back to unigenes. According to the mapping results, the quantification index of expression was calculated based on the formula of TPM (transcripts per million reads) by using RSEM v1.3.1 (University of Wisconsin-Madison, Madison, WI, USA) [62]. A R-based tool within the Bioconductor project, EdgeR v3.24.3 (Garvan Institute of Medical Research, Sydney, Australia) [63] was utilized to calculate log2(FC) and p-adjust value. When |log2(FC)| > 1 and p-adjust < 0.05, the unigenes were considered to be DEGs. In addition, functional-enrichment analysis of KEGG were performed using KOBAS to identify which DEGs were significantly enriched in metabolic pathways.

### 4.7. Gene Expression Validation and Structure Analysis

Relative quantification of DEGs were conducted using SYBR green I-based RT-qPCR assay, which was performed on LineGene 9600 Plus (Bioer Technology Co., Ltd., Hangzhou, China). As described in Section 4.4, total RNA was extracted in triplicate and RNA concentration and quality were evaluated. The removal of genomic DNA was performed under the PCR program set as 42 °C for 2 min in a 16 μL mixture consisting of 4 μL gDNA wiper Mix (Vazyme Biotech Co., Ltd., Nanjing, China), 500 ng total RNA, and RNase Free ddH_2_O. Then 4 μL qRT SuperMix II (Vazyme Biotech Co., Ltd., Nanjing, China) was added into the above reaction fluids and was used to reverse transcription with the condition set as 50 °C for 15 min and 80 °C for 2 min. Primers were designed using Primer Premier v5.0 software (PREMIER Biosoft, San Francisco, CA, USA) (Table S5) and synthesized by Sangon Biotech Co., Ltd., Shanghai, China. The *Ct60S* gene (KJ634810) was treated as a reference gene for normalization. Then 10 μL ChamQ SYBR Color qPCR Master Mix (Vazyme Biotech Co., Ltd., Nanjing, China), 0.4 μL primer F (5 μM), 0.4 μL primer R (5 μM), 2 μL template cDNA, and 7.2 μL ddH_2_O were mixed for PCR. PCR conditions comprised an initial holding at 95 °C for 5 min, and the cycle stage consisted of 95 °C for 30 s, 52 °C for 30 s, and 72 °C for 40 s for 40 cycles. The ∆∆Cq method was used to determine the relative abundance of DEGs [64]. Moreover, CDS prediction included two steps. First, unigenes were aligned according to the priority order of NR and Swissprot. If matched, the open reading frame (ORF) information was extracted from the results. If not, TransDecoder v5.5.0 (Minnesota Supercomputing Institute, Minneapolis, MN, USA) [65] was used to identify the candidate CDS region. Bcftools v1.9 (Genome Research Limited, Hinxton, UK) [66] was used to find the candidate SNP. In addition, MISA v2.3.6 [67] with default parameters was used to detect the SSR of unigenes.

### 4.8. Sequence Analysis of 4 CHS Unigenes

CHS is the entry enzyme in flavonoid biosynthesis and *CtCHS1* (KY471385) had been proved to play an important role in quinochalcone biosynthesis [36]. Consequently, we focused on 4 CHS unigenes selected from the six cDNA libraries of safflower. The deduced protein sequences of 4 CHS unigenes were analyzed using ExPASY [68]. Multiple sequence alignment was performed by using the DNAMAN v8.0 software (Lynnon Corporation, Quebec, Canada). The phylogenetic relationships of *CHS* genes from dissimilar plants were shown through a phylogenetic tree constructed by using a neighbor-joining method implemented with the MEGA X: Molecular Evolutionary Genetics Analysis across computing platforms (Kumar, Stecher, Li, Knyaz, and Tamura 2018) [69]. The parameters of the constructed trees were: phylogeny reconstruction: bootstrap method (1000 replicates), substitution model: amino acid and p-distance, substitutions to include: all, pattern among lineages: same (homogeneous), and rates among sites: uniform rates.

## 5. Conclusions

Previous studies have studied UDP-glycosyl-transferase, flavanone 3-hydroxylase, chalcone synthase, and chalcone isomerase of safflower. In this study, we found both the contents of SYP and SRP were higher in RYH than WXHH. In order to explain the formation mechanism of white florets, we first conducted transcriptome analysis of safflower with white and red florets and focused on the whole known biosynthesis pathway of SP. Furthermore, we identified 10 candidate unigenes related to safflower pigment biosynthesis, which may be helpful to produce SP. The huge unigenes generated in this study will be an invaluable resource for further studies involving functional genomics, molecular biology, metabolic engineering, and plant breeding of safflower.

## Figures and Tables

**Table 1:** The contents of SYP and SRP in the six samples.

Sample No.	Variety	Color	A_Y_	C_Y_ (g/g)	Average (C_Y_) (g/g)	A_R_	Average (A_R_)
W1	WXHH	White	0.125	0.0080	0.0079	0.108	0.110
W2	WXHH	White	0.121	0.0078		0.111	
W3	WXHH	White	0.124	0.0079		0.112	
R1	RYH	Red	0.876	0.0613	0.0588	4.000	3.337
R2	RYH	Red	0.889	0.0622		2.910	
R3	RYH	Red	0.753	0.0529		3.101	

WXHH, Xinhonghua No. 7; RYH, Yunhong No. 2; A_Y_, absorbance of safflower yellow pigment at 403 nm; C_Y_ (g/g), the content of SYP; Average (C_Y_) (g/g), the average C_Y_ of W1, W2, W3 and R1, R2, R3; A_R_, absorbance of SRP at 520 nm; Average (A_R_), the average A_R_ of W1, W2, W3 and R1, R2, R3.

**Table 2:** Summary of RNA-seq and assembly from WXHH and RYH.

	WXHH	RYH
Average Clean Reads	45,345,156	47,156,100
Average Clean Bases	6,793,392,182	7,063,377,560
Average Error Rate (%)	0.0229	0.0229
Average Clean Reads Q20 (%)	98.95	98.97
Average Clean Reads Q30 (%)	96.15	96.21
Average GC Content (%)	46.07	46.31
Total transcripts	304,392	
Total unigenes	225,008	
Average length (bp)	697.36	
N50 (bp)	1080	
GC percent (%)	42.32	

**Table 3:** Candidate genes related to safflower pigment biosynthesis

Function	Gene	Enzyme	KO Id (EC No.)	No.
Phenylpropanoid biosynthesis	*PAL*	Phenylalanine ammonia-lyase	K10775 (4.3.1.24)	7
	*4CL*	4-coumarate-CoA ligase	K01904 (6.2.1.12)	20
	*C4H*	Trans-cinnamate 4-monooxygenase	K00487 (1.14.14.91)	1
Flavonoid biosynthesis	*CHS*	Chalcone synthase	K00660 (2.3.1.74)	7
	*CHI*	Chalcone isomerase	K01859 (5.5.1.6)	4
	*F3H*	Flavanone 3-hydroxylase	K00475 (1.14.11.9)	1

KO, KEGG Orthology.

## Supplementary Materials

The following are available online at https://www.mdpi.com/2223-7747/9/7/847/s1: Figure S1: Volcano plots depict DEGs obtained by comparing WXHH with RYH. Up-regulated DEGs are indicated by red dots, down-regulated DEGs are indicated by green dots. DEGs that do not exceed the threshold of *p*-value < 0.05 & |log2(FC)| > 1 are depicted in grey. Figure S2: DEGs mapped to phenylpropanoid biosynthesis. All the products with color background in the figure belong to the background annotation result of this item. The blue border indicates the unigenes of whiteVSred_up, the red border indicates the unigenes of whiteVSred_down. Figure S3: DEGs mapped to flavonoid biosynthesis. All the products with color background in the figure belong to the background annotation result of this item. The blue border indicates the unigenes of whiteVSred_up, the red border indicates the unigenes of whiteVSred_down. Figure S4: Unrooted phylogram comparison of the amino acid sequences of 4 CHS unigenes and other functionally characterized CHS proteins download from NCBI. Table S1: Statistical table of comparison with assembly results. Table S2: Results of RT-qPCR. Table S3: Structure analysis of candidate unigenes. Table S4: Specific information of the six batches of the flowers of safflower. Table S5: Primers for RT-qPCR.

## Abbreviations

SP	safflower pigments
QCGs	quinochalcone C-glycosides
WXHH	Xinhonghua No. 7
RYH	Yunhong No. 2
PAL	phenylalanine ammonia-lyase
4CL	4-coumarate-CoA ligase
C4H	trans-cinnamate 4-monooxygenase
CHS	chalcone synthase
CHI	chalcone isomerase
F3H	flavanone 3-hydroxylase
DEGs	differentially expressed unigenes
SYP	safflower yellow pigments
SRP	safflower red pigments
HSYA	hydroxysafflor yellow A
RT-qPCR	reverse transcription-quantitative real-time PCR
CDS	coding sequence
SNP	single nucleotide polymorphisms
SSR	simple sequence repeats
KO	KEGG Orthology
TCM	Traditional Chinese Medicine
