# Supplementary material for: Transcriptome Sequencing and Chemical Analysis Reveal the Formation Mechanism of White Florets in Carthamus tinctorius L
Source: Plants (Basel). 2020 Jul 4;9(7):847. doi: 10.3390/plants9070847 (PMC7412316; doi:10.3390/plants9070847)
Supplement: Supplementary file 1 [file plants-09-00847-s001.zip › Supplementary Materials/Supplementary Figures.docx]

Supplementary Figures:





**Figure S1.** Volcano plots depict DEGs obtained by comparing WXHH with RYH. Up-regulated DEGs are indicated by red dots, down-regulated DEGs are indicated by green dots. DEGs that do not exceed the threshold of *p*-value < 0.05 & |log2(FC)| > 1 are depicted in grey.


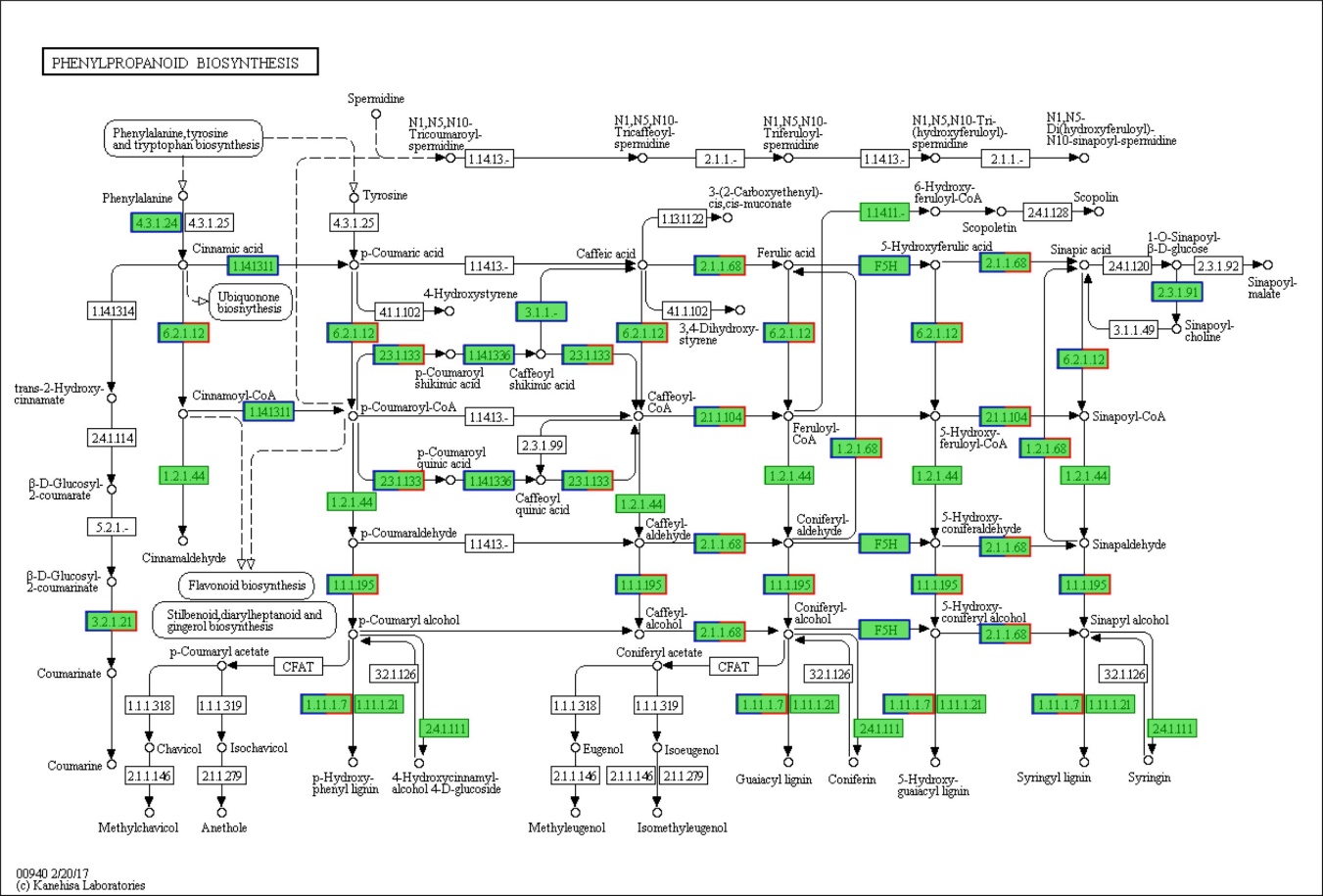


**Figure S2.** DEGs mapped to phenylpropanoid biosynthesis. All the products with color background in the figure belong to the background annotation result of this item. The blue border indicates the unigenes of whiteVSred_up, the red border indicates the unigenes of whiteVSred_down.


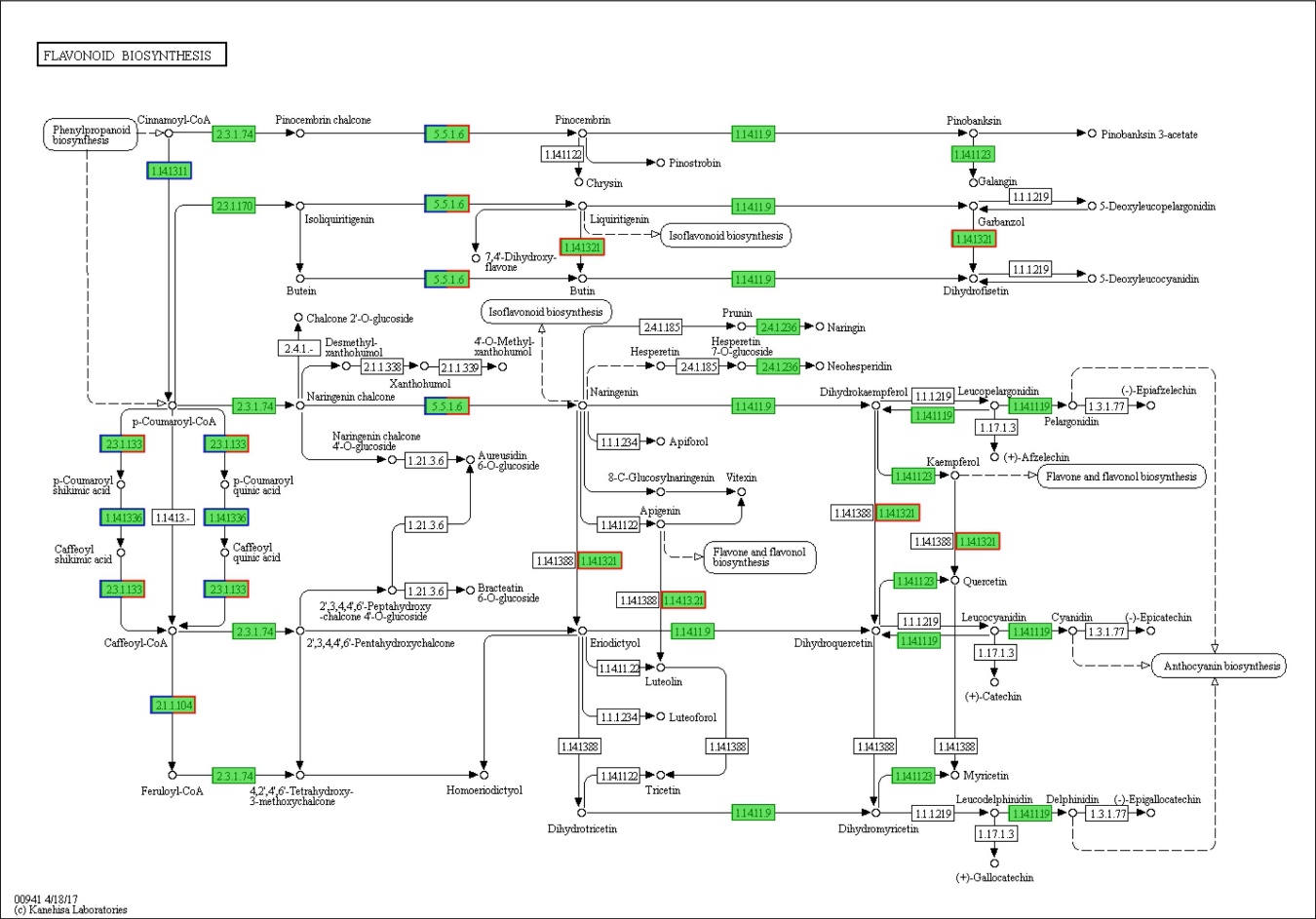


**Figure S3.** DEGs mapped to flavonoid biosynthesis. All the products with color background in the figure belong to the background annotation result of this item. The blue border indicates the unigenes of whiteVSred_up, the red border indicates the unigenes of whiteVSred_down.


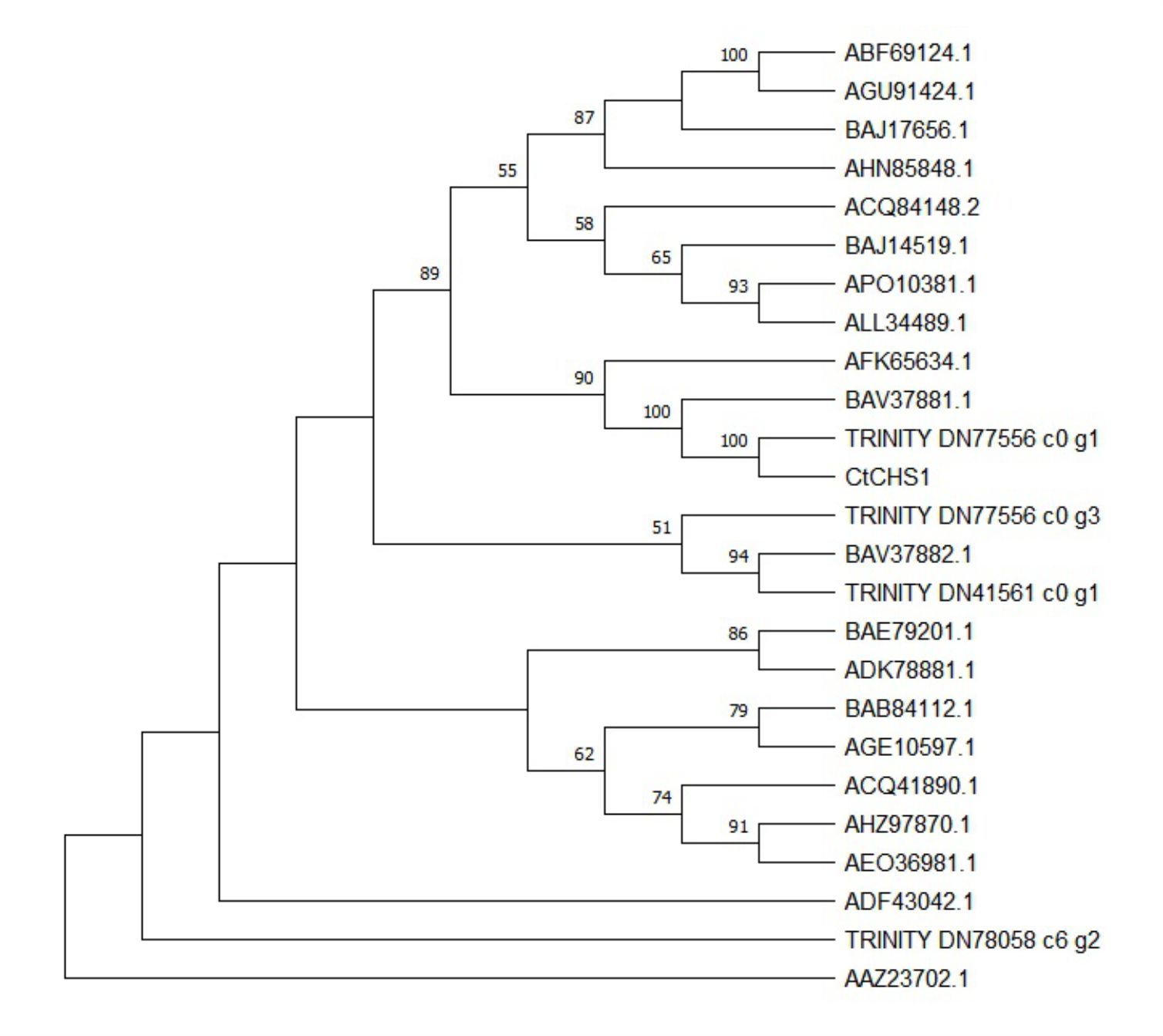


**Figure S4.** Unrooted phylogram comparison of the amino acid sequences of 4 CHS unigenes and other functionally characterized CHS proteins download from NCBI.
