# Supplementary figures and images for: Transcriptome Sequencing and Chemical Analysis Reveal the Formation Mechanism of White Florets in Carthamus tinctorius L
Source: Plants (Basel). 2020 Jul 4;9(7):847. doi: 10.3390/plants9070847 (PMC7412316; doi:10.3390/plants9070847)

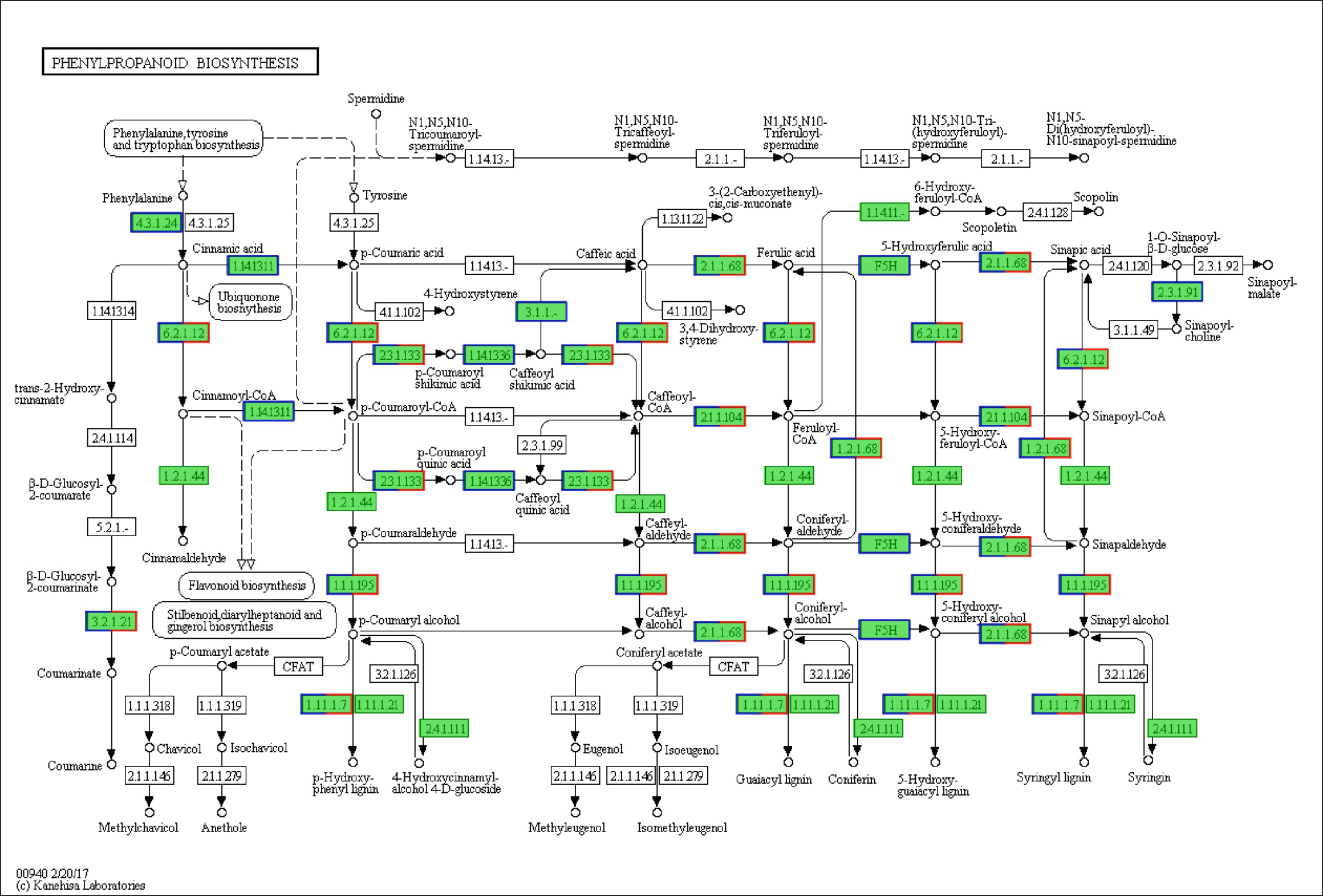

Supplement: Supplementary file 1 [file plants-09-00847-s001.zip › Supplementary Materials/Figure S2.jpg]

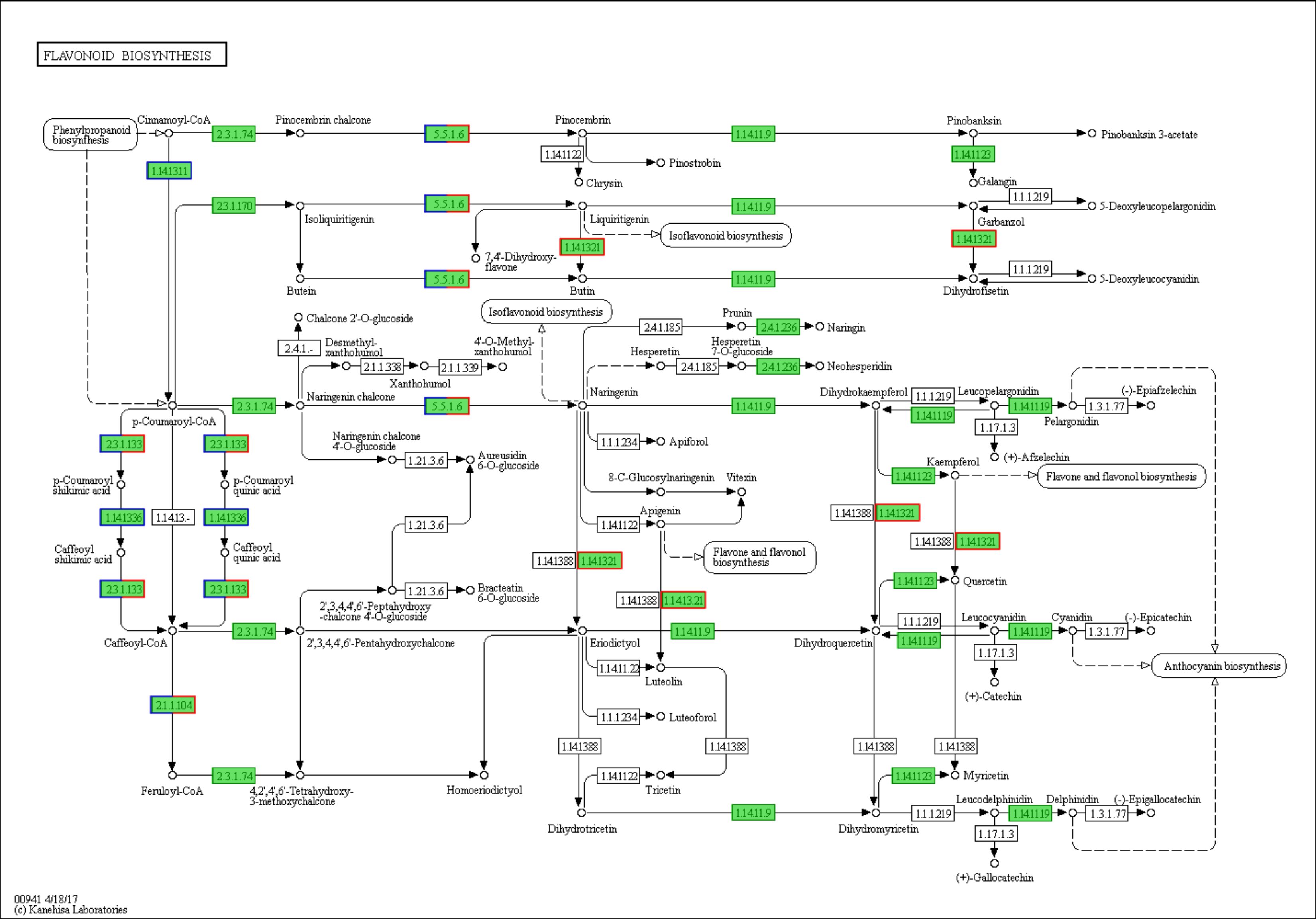

Supplement: Supplementary file 1 [file plants-09-00847-s001.zip › Supplementary Materials/Figure S3.jpg]

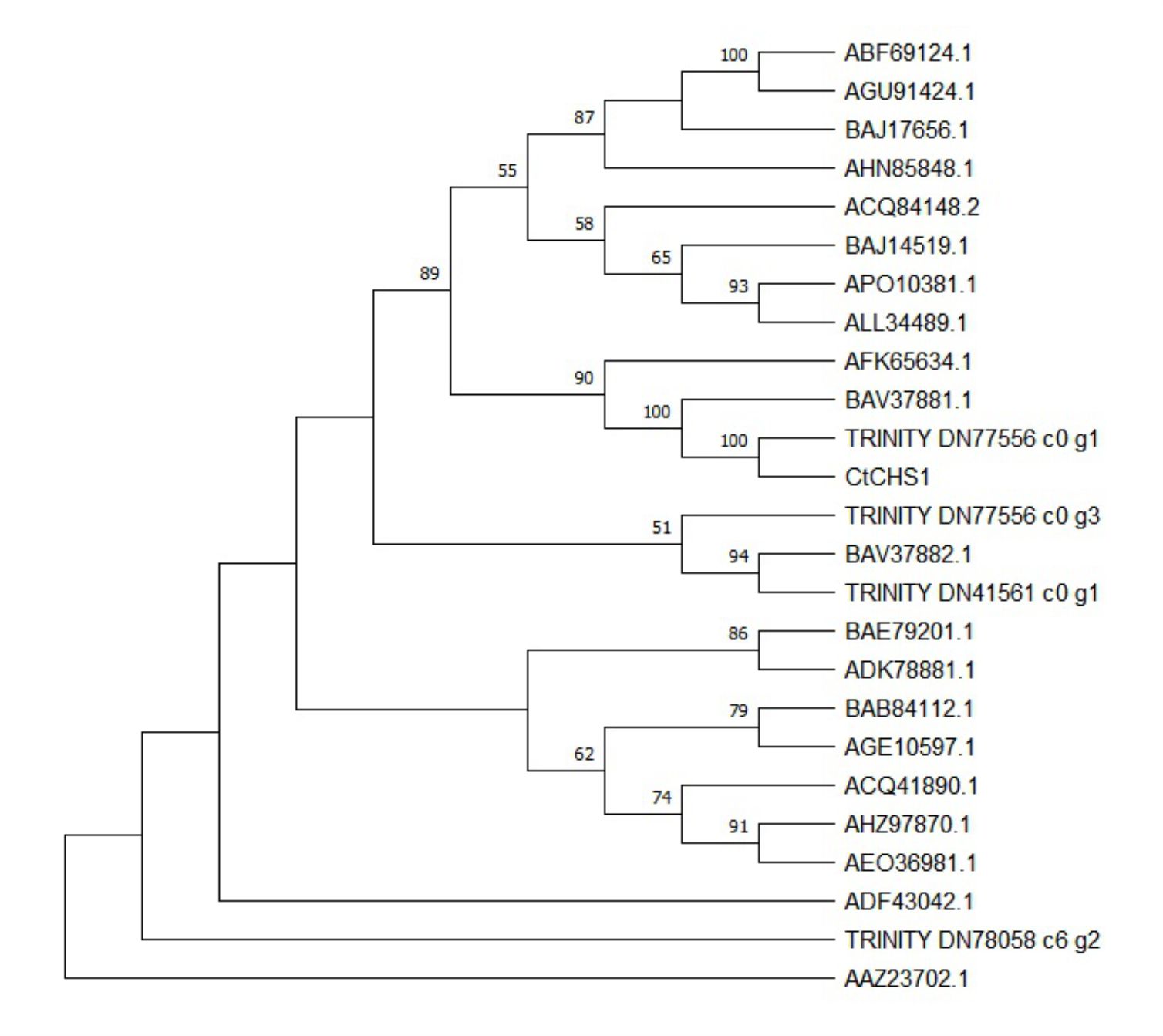

Supplement: Supplementary file 1 [file plants-09-00847-s001.zip › Supplementary Materials/Figure S4.jpg]
